# The impact of sodium fluoride addition to an adhesive system on micro tensile bond strength of ceramic occlusal veneer

**DOI:** 10.1007/s10266-026-01374-8

**Published:** 2026-04-13

**Authors:** Doaa Shawky Abdelmenam, Waleed Mohamed Elshahawy, Rania Khedr Ahmed

**Affiliations:** Department of Fixed Prosthodontics, Faculty of Dentistry, Tanta University, El-Geish Street, Tanta, 31511 Egypt

**Keywords:** Sodium fluoride (NaF), Adhesive systems, Deep dentin, Bond durability, Resindentin interface, Microtensile bond strength

## Abstract

This study aimed to assess the effect of incorporating sodium fluoride (NaF) into an adhesive system on the microtensile bond strength of ceramic occlusal veneers. Eighty resin–dentin sticks (~ 1 mm^2^) were obtained from 20 upper first premolars, with each tooth yielding four sticks. The teeth were sectioned using an Isomet saw to expose deep dentin, and Vita Enamic occlusal veneers were fabricated and cemented prior to sectioning. The specimens (n = 20) were assigned to two adhesive groups: a control (unmodified adhesive) group and a NaF-containing adhesive group. Each group was further subdivided into two subgroups, which were tested after 24 h and 6 months of storage in artificial saliva at 37 °C. Microtensile bond strength was measured using a universal testing machine. Repeated-measures ANOVA revealed no significant effect of adhesive type or storage duration on tensile bond strength. Independent *t* test showed a highly significant difference between subgroups I-B and II-B, with no significant differences in other comparisons. Mixed failures predominated across all groups, indicating balanced interfacial and cohesive properties, and EDX confirmed successful fluoride incorporation. Incorporation of sodium fluoride into the adhesive system did not alter immediate dentin bond strength but reduced long-term durability of ceramic partial-coverage restorations.

## Introduction

Minimally invasive indirect partial-coverage restorations, such as “occlusal veneers” provide an effective option for restoring posterior teeth affected by occlusal wear or advanced erosive lesions. Vita Enamic is a suitable material for these restorations, because its hybrid ceramic–polymer structure combines strength and elasticity, providing dentin-like stress distribution, high fracture resistance, and reliable bonding to resin cements. This supports conservative tooth preparation while maintaining durability under occlusal load [1].

The high tubule density and fluid content of deep dentin result in more fluid-filled spaces within the dentin, which can dilute adhesive monomers and interfere with proper hybrid layer formation. In addition, the increased fluid flow may promote adhesive hydrolysis over time thereby reducing the stability and durability of the bond [2, 3].

Bond degradation in deep dentin is further exacerbated by the activity of matrix metalloproteinases (MMPs), endogenous endopeptidases that physiologically remodel dentin. However, when reactivated during restorative procedures, MMPs can degrade collagen fibrils within the hybrid layer, which in turn significantly reduces long-term resin–dentin bond strength, compromises adhesive performance, and increases the risk of restoration failure [4, 5].

It is noteworthy to highlight the significance of sodium fluoride (NaF) as an MMP inhibitor. Although the selected NaF concentration (5000 ppm) is relatively high, it has been documented as safe and clinically acceptable with no significant adverse effects reported [6–8].

In addition, this level has been shown to inhibit matrix metalloproteinases (MMP-2 and MMP-9) in vitro, suggesting a potential mechanism by which high fluoride levels may influence collagen stability, although the exact pathways remain unclear as reported by Kato et al. [9].

Similarly, Samani et al. [10] demonstrated that the incorporation of the same concentration of NaF into a two-step etch-and-rinse adhesive system preserves immediate microtensile bond strength to dentin and enhances the long-term bond durability.

Accordingly, the objective of this study was to investigate the effect of sodium fluoride (NaF) incorporation into a two-step etch-and-rinse adhesive system on the microtensile deep dentin bond strength of Vita Enamic ceramic occlusal veneers. The null hypothesis of this study was that “there would be no difference in the bond strength when using conventional adhesive or adhesive containing NaF on the tested ceramic dentin micro tensile bond strength.”

## Materials and methods

This controlled comparative in vitro study was approved by The Research Ethics Committee (REC), Faculty of Dentistry, X University (FP3-242,157) and was performed at the Department of Fixed Prosthodontics, Faculty of Dentistry, X University.

Twenty sound upper first premolars were collected from outpatient clinics at the Oral and Maxillofacial Surgery Department after obtaining the donors' signed informed consent. Teeth were brushed with non-fluoridated pumice, rinsed thoroughly with tap water and stored in 0.9% sodium chloride solution with 0.1% thymol (Normal saline, Cairo, Egypt) [11]. The teeth were then mounted in acrylic blocks using a surveyor (Surveyor B2, BioArt, São Carlos, Brazil), with the level set 3 mm apical to the cementoenamel junction.

### Occlusal veneer preparation

**Fig. 1:**
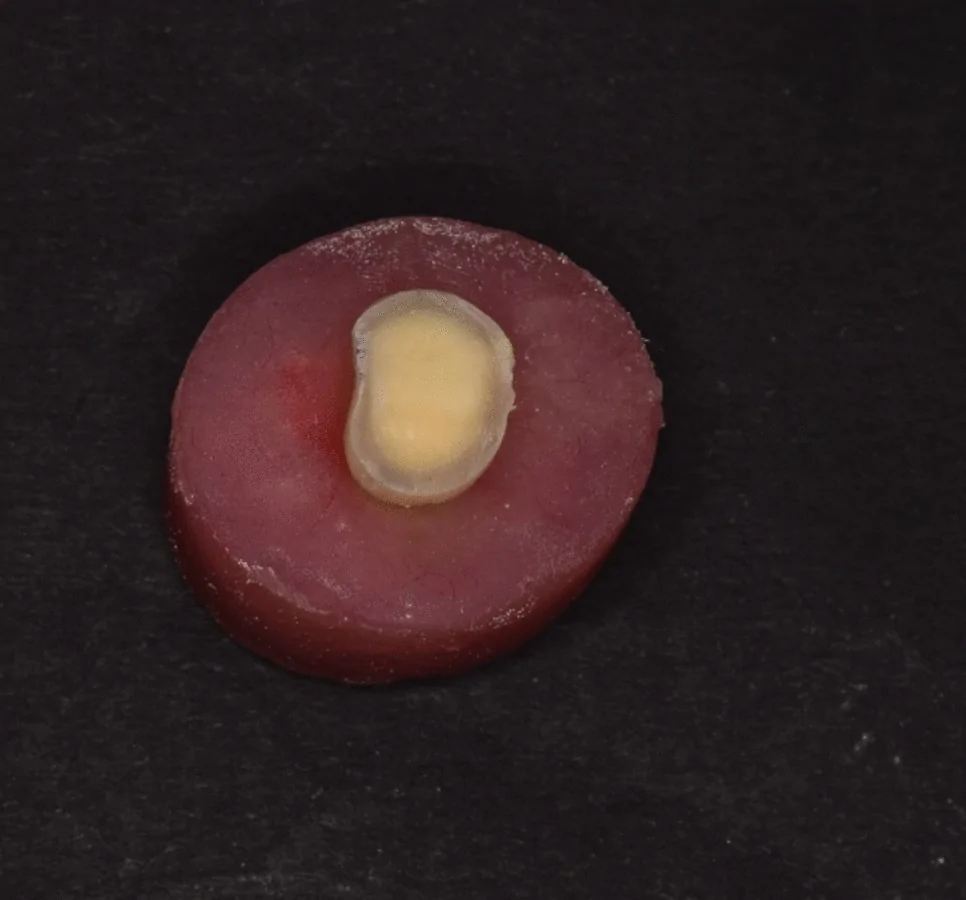
Flat occlusal surface after cutting by Isomet

After teeth had been mounted in acrylic blocks, standardized teeth preparations were accomplished using a linear precision saw (IsoMet 4000, Buehler; Lake Bluff, IL, USA), to obtain flat uniform surfaces. “All coronal tooth structure 4 mm occlusal to the cemento-enamel junction was removed, leaving exposed deep dentin centrally and enamel peripherally” (Fig. 1). In a pilot study, two designs were tested: one covering both enamel and dentin, and another leaving the peripheral enamel intact. The latter was selected to preserve occlusal surface integrity, ensure stable handling of the cemented veneers during sectioning, and reduce the number of specimens required.

### Specimen size calculation

Specimen size was calculated using G*Power software (version 3.1.9), targeting 80 specimens (resin–dentin sticks) from 20 teeth (experimental units). The experimental unit was considered to be the tooth rather than the individual sticks. Each tooth yielded four sticks, and their bond strength values were averaged to achieve a power of 96.8% with a significance level of 0.05 and a 95% confidence interval.

### Specimen grouping:

All teeth (*n* = 20) were randomly allocated to the groups using a computer-generated randomization list, then assigned as follows:

**Group I (control group):** included 10 teeth (*n* = 10) that received vita enamic (Vita, Zanhnfabrik, Germany) occlusal veneers bonded with the original adhesive without the addition of NaF. The group was further divided into:

- **Sub-group I-A (*n* = 5):** Was evaluated following a 24-h storage in artificial saliva at 37 °C.
- **Sub-group I-B (*n* = 5):** Were tested subsequent to 6 months of storage under the same conditions.

**Group II (test group):** Included 10 teeth (*n* = 10) that received vita enamic occlusal veneers bonded with NaF containing adhesive (5000 ppm NaF). This group was further subdivided into:

- **Sub-group II-A (*n* = 5) and sub-group II-B (*n* = 5):** Were tested after the identical storage periods and conditions as the control group.

### Scanning and design

Peripheral enamel was marked, and the prepared surfaces were coated with scanning spray (Digiscan-Spray, Yeti Dental Produkte). The teeth were then scanned (Freedom, DOF Inc., Seoul, Korea), and the data were saved as STL files [12]. Occlusal veneers were designed in Exocad (Exocad GmbH, Eiterfeld, Germany) and customized to the tooth anatomy with standardized thicknesses (1.5 mm at the fossa, 2 mm at the cusp) and a 0.05 mm cement gap. The STL files were milled from Vita Enamic blocks using a 5-axis CAD/CAM machine (imes-icore, Germany) [13].

### Adhesive preparation

The adhesive system used in this study is listed in (Table 1). For the NaF-containing formulation, 20 mg of NaF (5000 ppm) was weighed using an analytical balance (Citizen CY-204, India) and added to 4 mL of dentin adhesive in a clean beaker [10]. This concentration was selected based on its established use in experimental and clinical dentistry. It represents a relatively high yet controlled level that provides sufficient fluoride ions to produce measurable effects on material interactions while avoiding saturation or adverse structural changes. Thus, it offers a balance between efficacy and experimental sensitivity [9, 10].

**Table 1:** Manufacturer, commercial name, and composition of the adhesive system used in this study

Material	Manufacturer	Commercial name	Composition
Adhesive	MADESBA, S.A. SPAIN	Ventura uni bond2	Hydroxy ethyl methacrylate, urethane methacrylate, Glycerin dimethacrylate,hydroxy propyl methacrylate
Dentinetchant	MADESBA, S.A. SPAIN	Ventura	37% orthophosphoricacid solution

Mixing was performed under a custom dark box made from thick cardboard and black paper. The mixture was stirred with a magnetic stirrer (IKA Werke GmbH & Co. KG, Germany) at 2000 rpm for 10 min to ensure homogeneity. The NaF-containing adhesive was then transferred to a labeled bottle.

For quality control, the unpolymerized material was dispensed into a laser-cut Teflon mold (4 mm × 4 mm). The mold was covered with a Mylar strip and a glass slide and then light-cured (Woodpecker, Guilin, China) for 20 s [14]. The specimens were polished (Sof-Lex, 3 M ESPE) and subsequently analyzed using a scanning electron microscope (SEM) equipped with energy-dispersive X-ray spectroscopy (EDX) (JEOL–SEM IT200, Tokyo, Japan). This analysis assessed mixture homogeneity and fluoride distribution, and confirmed proper NaF incorporation.

### Restorations luting procedures

Vita Enamic veneers were etched with 5% hydrofluoric acid for 60 s, rinsed, dried, and silanized using two coats of Bisco Porcelain Primer (Bisco, Inc., Schaumburg, IL, USA). Dentin was etched with 37% phosphoric acid for 15 s, rinsed, dried, and bonded with either the original or NaF-containing adhesive, followed by light curing for 10 s [15]. Dual-cure resin cement (Duo-Link, dual-cure resin cement, Bisco, Inc., Schaumburg, IL, USA) was then applied, and the veneers were seated under a constant load of 49 N (equivalent to 5 kg) for 5 min using a universal testing machine (Instron 3345 universal testing machine) to standardize pressure during cementation. Excess cement was removed, and the restorations were light-cured for 30 s [16].

### Resin–dentin stick preparation

**Fig. 2:**
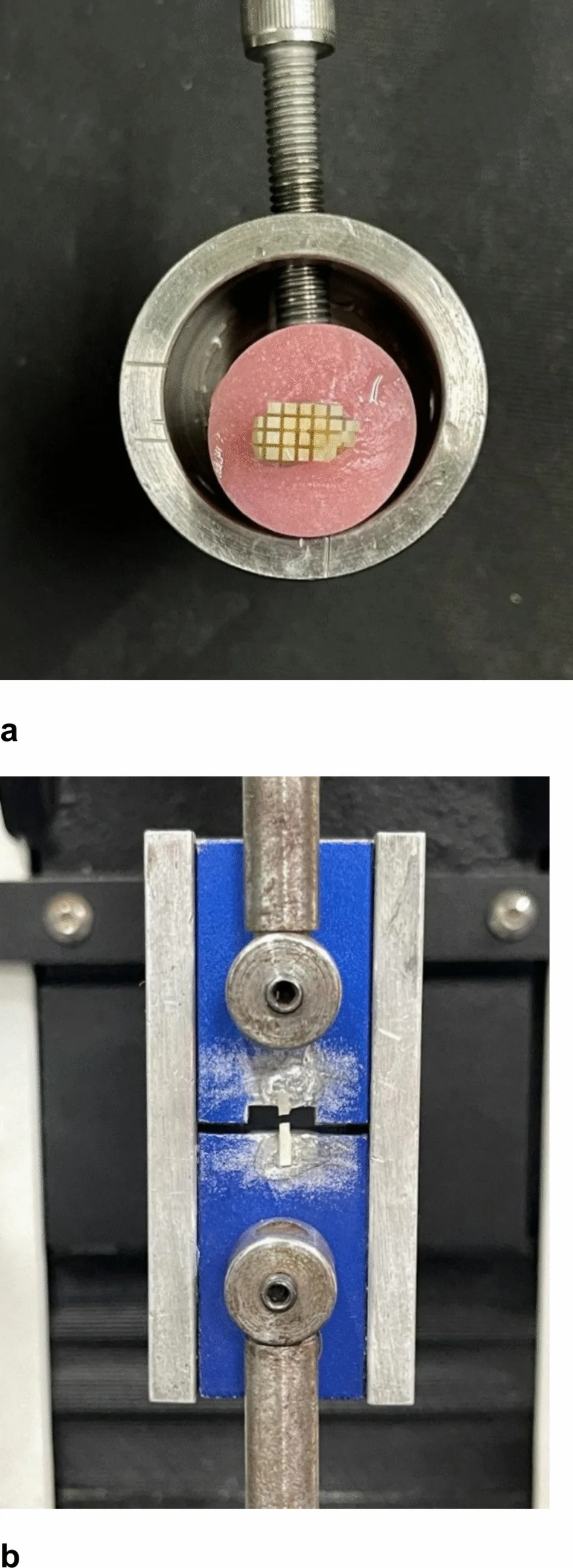
Microtensile bond strength test. **a** Isomet cutting of the cemented occlusal veneer buccolingually and mesiodistally. **b** Application of microtensile force after attaching the beam by a cyanoacrylate adhesive

The specimens were cut longitudinally in both mesiodistal and buccolingual orientations to full depth across the adhesive interface using an Isomet saw (Fig. 2a) under copious irrigation to obtain beams. These beams were further trimmed to produce four resin–dentin sticks from each tooth: three from the center and one located 0.5 mm from the margin, each with an adhesive surface area of approximately 1 mm^2^ [17]. A digital caliper was used to measure the cross-sectional area of each stick. The resin–dentin sticks from each subgroup then underwent microtensile bond strength testing after storage in artificial saliva at 37 °C for either 24 h or six months. Artificial saliva was used as the storage medium to better simulate intraoral conditions and the oral environment [18]. It was replenished daily to maintain consistency [19].

### Microtensile bond strength test

The operator performing the microtensile bond strength testing and failure mode analysis was blinded to group allocation. Each stick was subsequently mounted on a jig for microtensile testing using a cyanoacrylate adhesive and subjected to tensile loading in a universal testing machine at a crosshead speed of 0.5 mm/min (Fig. 2b). Data were calculated and recorded using computer software.

### Failure mode analysis

The fractured specimens were examined by a single evaluator under a stereomicroscope (× 40) and classified as adhesive (at the adhesive–dentin or adhesive–ceramic interface), cohesive (within dentin or ceramic), or mixed (involving both the interface and the substrate).

### Statistical analysis

Data were analyzed using SPSS v26. The mean bond strength value per tooth was calculated and used as the statistical unit to avoid pseudo-replication. Numerical data were expressed as mean ± SD and range, while categorical data were expressed as frequency and percentage. The normality of data distribution was assessed using the Shapiro–Wilk test. The effect of adhesive type (between-subject factor) and storage duration (within-subject factor) on tensile bond strength was evaluated using repeated-measures ANOVA. In addition, pairwise comparisons between independent groups (Group I vs Group II) were performed using independent *t* test. All results are reported as mean ± standard deviation (SD), with *P* < 0.05 (*) considered significant and *P* < 0.001 (**) considered highly significant.

## Results

The tensile bond strength values of the tested groups (Table 2) were analyzed using repeated-measures ANOVA. No statistically significant effect of adhesive type on tensile bond strength was observed in subgroup A (*F* = 3.583, *P* = 0.131) or subgroup B (*F* = 2.362, *P* = 0.199). Similarly, no significant effect of storage duration (Table 3) was found when comparing subgroups A and B (*F* = 0.010, *P* = 0.921).

**Table 2:** Maximum tensile bond strength (MPa) of Groups I and II in subgroups A and B (independent *t* test and repeated-measures ANOVA)

Tensile stress at Maximum (MPa)		Type of adhesive						Independent *t* test	
		Group I			Group II			t	*p* value
Subgroup A	Range	6.29	–	12.19	5.61	–	13.63	−0.546	0.600
	Mean ± SD	9.006	±	2.500	9.982	±	3.117		
Subgroup B	Range	9.38	–	15.25	2.56	–	10.24	3.080	0.015*
	Mean ± SD	12.706	±	3.043	6.650	±	3.174		
Repeated-measures ANOVA	F	3.583			2.362				
	*p* value	0.131			0.199				

^*^ Statistically significant difference at *p * ≤ 0.05

**Table 3:** Maximum tensile bond strength (MPa) at different storage durations in subgroups A and B analyzed by repeated-measures ANOVA

Tensile stress at Maximum (MPa)	Durations						Repeated Measure ANOVA	
	Subgroup A			Subgroup B			F	*p* value
Range	5.61	–	13.63	2.56	–	15.25	0.010	0.921
Mean ± SD	9.494	±	2.713	9.678	±	4.334		

Independent *t* tests (Table 2) showed no significant difference between Group I and Group II in subgroup A (*t* = −0.546, *P* = 0.600), whereas subgroup B exhibited a statistically significant difference (*t* = 3.080, *P* = 0.015), with Group I-B displaying higher tensile bond strength than Group II-B.

The distribution of failure modes was analyzed using the chi-square test (Table 4). In subgroup A, the differences in the frequencies of cohesive, adhesive, and mixed failures between the two adhesives were not statistically significant (*p* = 0.435). Similarly, in subgroup B, no significant differences were observed between the two adhesives in failure mode distribution (*p* = 1.000). Overall chi-square analysis across durations and adhesive types also showed no significant association between failure mode and either adhesive type or storage duration (*p* = 0.766 and *p* = 0.368, respectively).

**Table 4:** Distribution of failure modes according to adhesive type and storage duration (chi-square test)

Durations	Mode of failure	Type of adhesive				Chi-square	
		Group I		Group II			
		N	%	N	%	X^2^	*p* value
Subgroup A	Cohesive	2	40.00	1	20.00	1.667	0.435
	Adhesive	1	20.00	3	60.00		
	Mixed	2	40.00	1	20.00		
Subgroup B	Cohesive	1	20.00	1	20.00	0.000	1.000
	Adhesive	1	20.00	1	20.00		
	Mixed	3	60.00	3	60.00		
Chi-square	X^2^	0.533		2.000			
	*p* value	0.766		0.368			

**Fig. 3:**
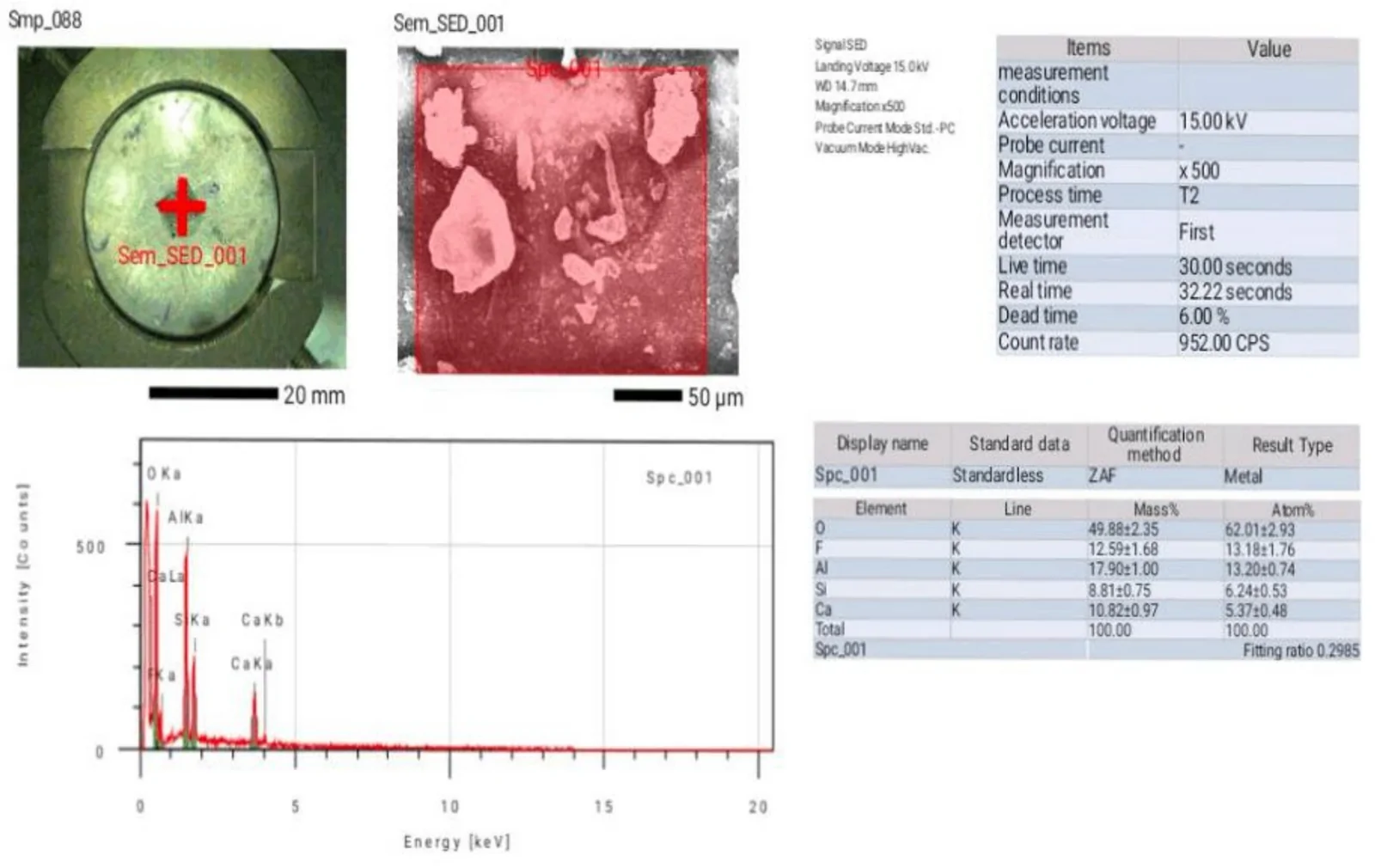
Representative SEM–EDX chart of sodium fluoride (NaF)-modified adhesive test piece

Representative EDX spectra and elemental distribution of the NaF-containing adhesive sample are shown in Fig. 3. Quantitative EDX analysis (Table 5) confirmed the presence of five elements in the NaF-containing adhesive sample. Fluorine was clearly incorporated (12.59 wt%, 13.18 at%) alongside oxygen, aluminum, silicon, and calcium. The total elemental composition was 100%, with a ZAF fitting ratio of 0.2985. These findings indicate that the adhesive retained its typical composition while successfully incorporating fluoride.

**Table 5:** EDX elemental analysis of the sodium fluoride (NaF)-containing adhesive sample

Element	Line	Mass % (wt.%)	Atomic % (at.%)
O	K	49.88 ± 2.35	62.01 ± 2.93
F	K	12.59 ± 1.68	13.18 ± 1.76
Al	K	17.90 ± 1.00	13.20 ± 0.74
Si	K	8.81 ± 0.75	6.24 ± 0.53
Ca	K	10.82 ± 0.97	5.37 ± 0.48
Total	–	100.00	100.00

## Discussion

The results support rejection of the null hypothesis, as a highly significant difference was observed between subgroups I-B and II-B, indicating superior long-term performance of the original adhesive and suggesting interfacial maturation and stabilization.

Repeated-measures ANOVA was performed to evaluate the effects of adhesive type and storage duration on tensile bond strength. The lack of a significant overall effect may be due to the nature of repeated-measures ANOVA, which assesses overall effects across all timepoints and groups. As a result, localized differences between specific subgroups may not reach statistical significance.

Therefore, an independent *t* test was subsequently performed to allow direct pairwise comparisons between the corresponding subgroups of adhesive type, which may not have been detected by the overall repeated-measures ANOVA.

A statistically significant difference was observed between subgroups I-B and II-B, indicating that adhesive composition becomes influential only after aging. This may be explained by environmental or time-dependent factors that accentuate structural or chemical differences between formulations, potentially making the modified adhesive more susceptible to hydrolytic degradation, reduced cross-link stability, or altered resin–dentin interactions [20].

This finding may also be partially attributed to the interaction between the adhesive system and the dual-cure resin cement. Although both groups were bonded using a two-step etch-and-rinse adhesive protocol, modification of the adhesive with NaF may have altered its chemical composition and interfacial characteristics. Such compositional changes could influence the compatibility between the adhesive layer and the dual-cure resin cement, potentially affecting polymerization efficiency at the adhesive–cement interface [10, 21].

It has been reported that residual acidic components within adhesive layers may interact with the amine initiators of chemically or dual-cured resin cements, which may interfere with polymerization and compromise the stability of the bonded interface [22]. Furthermore, storage in artificial saliva may enhance water sorption and hydrolytic degradation over time, which could further accentuate differences in bond durability between the original and the NaF-containing adhesive formulations [18, 19].

The absence of a statistically significant difference between the two adhesive types in subgroup A indicates that, under this condition, both formulations exhibited comparable mechanical performance. This suggests that the immediate bonding effectiveness of the NaF-modified adhesive is similar to that of the conventional adhesive, implying that fluoride incorporation did not adversely affect early polymerization, hybrid layer formation, or initial interfacial integrity. In practical terms, both systems appear equally capable of establishing an adequate micro-mechanical bond shortly after application. This reinforces the interpretation that adhesive performance is governed by multifactorial interactions rather than by material composition or aging time alone [2, 23].

Within each adhesive, the absence of significant differences between subgroups A and B indicates that storage duration alone did not independently affect bond strength. However, Group I showed a near-significant trend toward higher strength in Subgroup B, which may reflect post-curing maturation, whereas Group II remained relatively stable, suggesting more time-independent behavior [24].

The findings of Costa et al. [25] are partially consistent with the present study. They reported no significant difference in dentin bond strength between fluoride-releasing and conventional adhesives under different aging conditions, which aligns with our immediate results showing comparable bond strength between the materials. However, unlike their study, our results demonstrated a material-dependent aging effect, as the NaF-containing adhesive showed reduced bond strength after 6 months, whereas the original adhesive did not.

El-Deeb et al. [23] showed partial agreement with the present findings. Their results were consistent with the immediate bond strength outcomes of this study but differed from the long-term results, as they reported that fluoride incorporation did not affect bond strength even after 6 months of aging. These discrepancies may be attributed to differences in adhesive formulation, fluoride incorporation methods, aging protocols, dentin substrate, or specimen preparation.

In contrast, some studies have reported results that differ from those of the present study. For example, Samani et al. [10] evaluated NaF-containing adhesives at two concentrations (5000 ppm and 10 000 ppm) and reported higher bond strength than unmodified controls at both 24 h and 3 months. This discrepancy may be attributed to variations in NaF concentration, adhesive formulation, or aging conditions.

Shinohara et al. [26] also investigated the durability of a commercial fluoride-containing adhesive and reported that although its bond strength was lower than that of non-fluoride adhesives at 24 h, it increased after 3 months of water storage, suggesting improved long-term bonding performance. This discrepancy may be attributed to differences in adhesive formulation, fluoride release, and storage media.

Moreover, the findings of Nikaido et al. [27] do not align with the present study’s results regarding long-term bond performance. Their results indicated that fluoride may enhance interfacial durability, which they attributed to the formation of a thicker and more protective acid–base resistant zone (ABRZ) that increases resistance to degradation.

The predominance of mixed failures is commonly associated with relatively balanced interfacial and cohesive properties, where fracture propagates through multiple regions rather than through a single weak interface. Furthermore, the absence of significant changes within each group over time suggests that storage duration and storage medium influenced bond strength values without substantially altering the underlying failure mechanism. This implies that the observed differences between groups are primarily quantitative rather than qualitative.

The EDX analysis validated the presence of both organic and inorganic components in the modified adhesive system, particularly highlighting the successful incorporation of fluoride.

This in vitro study was limited by its 6-month artificial saliva storage, the use of a single two-step etch-and-rinse adhesive, and the absence of oral dynamic simulations or evaluation of fluoride release, dentin penetration, and long-term hybrid layer interactions.

Future studies should assess the long-term performance of NaF-containing adhesives through extended in vitro and clinical trials, considering variables, such as bruxism, diet, and oral hygiene. Researchers could evaluate different adhesive systems, NaF concentrations, and aging protocols, alongside nanoleakage or microscopic analyses to clarify the mechanisms of bond reinforcement.

## Conclusion

In this in vitro study, NaF-modified adhesive showed similar immediate dentin bond strength to the conventional adhesive. However, after 6 months, its bond durability was lower than that of the original adhesive. Mixed failures predominated, and EDX confirmed fluoride incorporation, highlighting the need for further long-term in vitro and clinical investigations.

## Data Availability

Data supporting the findings of this study are available from the corresponding author upon request.
